# Disaster literacy and preparedness among nursing students in earthquake-affected Turkey: a cross-sectional study

**DOI:** 10.1186/s12912-026-04975-4

**Published:** 2026-07-07

**Authors:** Türkan Karaca, Nilay Ercan Sahin

**Affiliations:** 1 Faculty of Health Sciences, Harran University, Nursing Harran University-Osmanbey Campus Haliliye, Şanlıurfa, Turkey; 2 Nursing Faculty, Hacettepe University-Altındag, Hacettepe University, Centre, Ankara, Turkey

**Keywords:** Natural disaster literacy, Disaster preparedness, Nursing students

## Abstract

**Aim:**

This study aims to assess nursing students’ natural disaster literacy and disaster preparedness levels and to explore the relationship between these factors.

**Methods:**

This cross-sectional study was conducted in the nursing department of a health sciences faculty at a university located in a province affected by the earthquake in southeastern Türkiye.The sample of the study consisted of 302 students. Data were collected using a demographic characteristics. form, the Natural Disaster Literacy Scale and the Disaster Preparedness Scale.

**Results:**

The mean total score for the students on the Natural Disaster Literacy Behavior Scale was found to be medium level as 36.54 ± 4.59. The mean total score for the students on the Natural Disaster Literacy Affective Disposition Scale was found to be high level as 47.75 ± 4.22. The mean total score for the students on the Disaster Preparedness Scale was found to be 27.85 ± 3.40. There is no statistically relationship was found between nursing students’ Natural Disaster Literacy and Disaster Preparedness.

**Conclusions:**

The conclusion of the study revealed that while students exhibited a moderate level of natural disaster literacy behaviors, their affective tendencies toward natural disaster literacy were high. The students’ average total score on the disaster preparedness scale was slightly above the scale’s overall mean score. There was no correlation identified between nursing students’ knowledge of natural disasters and their level of disaster preparedness. Knowledge and attitude cultivation alone seldom converts into concrete disaster-prevention actions. Going forward, nursing education should build on theoretical coursework by systematically integrating scenario-based simulations, hands-on drills, and interprofessional collaboration. Such measures will progressively strengthen students’ practical skills and emergency decision-making abilities, thereby comprehensively enhancing their effectiveness in real-world disaster situations.

**Clinical trial number:**

Not applicable.

## Introduction

Natural disasters are events that cause physical, psychological, environmental, economic, and social losses, varying in duration and intensity, which halt or disrupt life, and for which the capacity of communities to cope and intervene is insufficient [1]. Natural disasters often begin suddenly and can result in consequences in a short period of time, making prevention difficult. According to the Emergency Events Database, initiated by the Centre for Research on the Epidemiology of Disasters, 399 disaster events occurred worldwide in 2023, resulting in the loss of 86,473 lives and negatively affecting approximately 93.1 million people [2, 3].

In the process of preparing for natural disasters, combating them, and reducing the damages caused by them, the literacy level of the community plays an important role. Increasing the natural disaster literacy level of individuals or communities contributes to the response to disasters, the ability to analyze and evaluate situations, maintaining objectivity, finding practical solutions to problems, and making correct decisions [4]. Additionally, it is believed that material and moral damages caused by natural disasters can be reduced with a higher level of natural disaster literacy [5]. Individuals with high natural disaster literacy possess knowledge about disasters; they know how to combat them, participate in volunteer activities, analyze outcomes, and warn others about secondary hazards in the environment [6]. It is anticipated that a person’s high level of disaster literacy will also increase their disaster preparedness by utilizing the knowledge they have gained [4].

Disaster preparedness is a crucial factor in reducing loss and destruction during all disaster situations [7]. Disaster preparedness encompasses all the measures that individuals and communities need to take in advance to minimize the harm caused by disasters. Preparedness, in a sense, refers to being ready, and includes secondary planning, implementation, and evaluation aimed at reducing risks and the destruction they cause [8].

A review of the literature reveals a limited number of studies examining nursing students’ natural disaster literacy and disaster preparedness levels. The results of a study by Şekerci and colleagues (2023) showed that disaster education positively influenced nursing students’ attitudes and behaviors regarding natural disaster literacy [9]. In a study by Şahin and colleagues (2018), it was found that students had low levels of preparedness for disasters [10]. However, a study conducted by Ertuğrul and Ünal (2020) found that students’ preparedness levels for disasters were above average [11]. These two concepts complement each other; disaster literacy provides the awareness and knowledge foundation necessary for individuals to be prepared for disasters, while disaster preparedness turns this knowledge into real experience and applies it in practice.

Nurses play a crucial role in disaster management, contributing to preparation, protection, intervention, and post-disaster recovery. In disaster situations, nurses are actively involved in initial intervention, rapid assessment, triage, and care [9]. For nursing students to fulfill these key roles in the future, it is essential that their natural disaster literacy and disaster preparedness levels are high.

The relationship between natural disaster literacy and disaster preparedness is assumed to be based on theoretical approaches suggesting that knowledge and awareness guide behavior. Individuals with greater knowledge about disasters are thought to better assess risks and, accordingly, are more likely to engage in preparedness behaviors. In this regard the instruments used in this study assess the dimensions of knowledge/awareness (literacy) and behavior (preparedness), making the examination of the relationship between these two variables theoretically meaningful. Furthermore, it is believed that conducting a study examining the relationship between disaster literacy and disaster preparedness among nursing students will help determine their levels and fill this gap in the literature. It is believed that the results of this study will provide guidance for disaster education to be given to university students, the aim of this research is to determine the natural disaster literacy and disaster preparedness levels of nursing students and to examine the relationship between these two factors.

## Method

### Research type

This research is a cross-sectional study conducted to determine the natural disaster literacy and disaster preparedness levels of nursing students and to examine the relationship between these two factors.

### Participants and setting

The research was conducted at the nursing department of a faculty of health sciences at Harran university located in a province affected by the earthquake in southeastern Turkey. The population of the study consisted of 352 nursing students enrolled in the 3rd and 4th Year of the nursing department at the faculty of health sciences during the fall semester of the 2024–2025 academic year. The sample inclusion criteria are being a third or fourth-year nursing student and being willing to participate in the study. No sampling method was used in the study, the aim was to reach all 352 students during the data collection process, 340 nursing students came to information meeting and 302 nursing students participated in the study who voluntarily agreed to participate in the study and completed the data collection tools. A power analysis was performed for this study using G*Power. For the independent samples t-test, the effect size was set as Cohen’s d = 0.5, α = 0.05, and power = 0.91.

### Data collection tools

In this study, data were collected using a demographic characteristics form, the Natural Disaster Literacy Scale (Behavioral and Affective Disposition), and the Disaster Preparedness Scale.

The demographic characteristics form consists of 10 questions designed to determine the students’ demographic characteristics. This form assesses students’ socio-demographic features, general knowledge about disasters (such as their experience with disasters, participation in disaster-related courses, and the names of disaster-related organizations, etc.).

The Natural Disaster Literacy Scale consists of two sections. The *Natural Disaster Literacy Affective Disposition Scale* (DAO Affective Disposition), developed by Sözcü and Aydınözü (2019), is aimed at determining individuals’ affective literacy regarding natural disasters. The scale consists of 25 items [4]. The scale includes three sub-dimensions: " susceptibility " (15 items), “awareness” (7 items), and “individual and societal preparedness” (3 items). The first 16 items of the scale are positive, and the last nine items are negative statements. Negative items are reverse-coded. The scale is a five-point Likert-type scale, with responses coded as “strongly agree, agree, undecided, disagree, strongly disagree.” The Cronbach’s alpha coefficient of the scale has been reported as 0.81. In this study, the Cronbach’s alpha coefficient was found to be 0.80.

The Natural Disaster Literacy Behavior Scale (DAO Behavior) is a scale developed by Sözcü and Aydınözü (2019) to determine individuals’ behavioral literacy in relation to natural disasters. The scale consists of 23 items [4]. The scale includes three subdimensions: “geographic inquiry (10 items),” " personal protection measure (6 items),” and " physical and ideological attitudes (7 items).” The first 16 items are positively worded, while the last seven items are negatively worded. Negative items are reverse coded. The scale is a five-point Likert type, coded as “always, mostly, sometimes, rarely, never.” The Cronbach’s alpha coefficient of the scale was reported as 0.87. In this study, the Cronbach’s alpha coefficient was found to be 0.82.

In addition, natural disaster literacy can be divided into three levels (low-medium-high). Scores ranging from 12 to 27 on both scales indicate “low,” scores from 28 to 44 indicate “medium,” and scores from 45 to 60 indicate “high” levels of natural disaster literacy [4].

The Disaster Preparedness Scale, developed by Şentuna and Çakı in 2020, is a scale designed to assess individuals’ preparedness for disasters. It consists of 13 items and 4 subdimensions (disaster physical protection, disaster planning, disaster assistance, and disaster warning systems) (Şentuna & Çakı, 2020). The scale is scored using a four-point Likert type, with total scores ranging from 13 to 52. An increase in the total score indicates a higher level of disaster preparedness. The Cronbach’s alpha value of the scale is 0.82 [8]. For the sample in this study, the Cronbach’s alpha value was found to be 0.83.

### Data collection

The research was conducted during the first week of the relevant fall semester of the students’ academic year (September 26–30, 2024). Before the data collection tools were applied, the first author gathered the students (340) in a single class before beginning data collection and provided the students with information about the study and emphasized that participation in the research was based on the principle of voluntariness. Students who agreed to participate in the study (302) were first asked to complete an informed written consent form. After obtaining the informed written consent form from the students, they were simultaneously given the Demographic Information Form, the Natural Disaster Literacy Scale (Behavioral and Affective Disposition), and the Disaster Preparedness Scale. The students were instructed to complete the relevant forms accurately and thoroughly. During the data collection, checks were conducted, and it was found that all forms were completed correctly and fully, with no missing data. The data collected by author (who is not have the teaching, supervisory, or evaluative role with the participating students) and collection process took approximately 15–20 min in one classroom setting.

### Data analysis

In the study, statistical analyses were performed using the Statistical Package for the Social Sciences for Windows 25 statistical software. In the analysis of the scales scores, measures such as mean, standard deviation, minimum, maximum were used. The relationship between the Natural Disaster Literacy Scale and Disaster Preparedness Scale scores were examined using Pearson Correlation Analysis.

### Ethics approval and consent to participate

To conduct the study, permission was obtained from the Social and Humanities Research Ethics Committee of the Harran University (2024/19). Written permission to conduct the research was also obtained from the Department of Nursing, Faculty of Health Sciences, at the relevant university. In addition, after the students were informed about the research, their written and verbal consent was obtained voluntarily who agreed to participate in the study. It was specified that students should not write their names on the questionnaire, ensuring the anonymity of the participants. The principles of the Declaration of Helsinki and publication ethics were followed in our study.

## Results

When examining the demographic characteristics of the students who participated in the study, it was found that 53.6% of the students were in their fourth year, and the majority were female (68.5%). Most students (88.4%) had not taken any courses related to disasters. All students had experienced a disaster in the past five years, and the majority (71.9%) had not participated in any disaster-related activities. More than half of the students (59.9%) were aware of disaster-related organizations. Only half of the participants (50.0%) knew the emergency assembly area on campus (Table 1).

**Table 1:** Demographic characteristics of students

Demographic Characteristics (*n* = 302)		Number		Percentage
Class				
3rd Year		140		46.4
4rd Year		162		53.6
Gender				
Female		207		68.5
Male		95		31.5
Disaster-related course participation				
Yes		35		11.6
No		267		88.4
Experience with a disaster in the last 5 years*				
Yes		302		100.0
No		0		0.0
Participation in disaster-related activities**				
Yes		85		28.1
No		217		71.9
Awareness of disaster-related organizations				
Yes			181	59.9
No			121	40.1
Knowledge of emergency assembly area on campus				
Yes			151	50.0
No			151	50.0
Total			302	100

*All students have experienced an earthquake disaster

**Activities include seminars, conferences, fire drills, and earthquake drills related to disasters

Table 2 presents the descriptive statistics of the students’ scores on the Natural Disaster Literacy and Disaster Preparedness scales, as well as their subscales. The mean total score for the students on the Natural Disaster Literacy Behavior Scale was found to be 36.54 ± 4.59. The mean total score for the students on the Natural Disaster Literacy Affective Disposition Scale was found to be 47.75 ± 4.22. The mean total score for the students on the Disaster Preparedness Scale was found to be 27.85 ± 3.40 (Table 2).

**Table 2:** Descriptive statistics of the students’ naturel disaster literacy scale and disaster preparedness scale (*n* = 302)

NDL Behavior Subscales	X ± SD
Geographic Inquiry	15.00 ± 3.03
Personal protection measure	7.11 ± 1.21
Physical and ideological attitudes	14.42 ± 2.02
NDL Behavior Scale Total Score	36.54 ± 4.59
NDL Affective Disposition Subscales	
Susceptibility	29.54 ± 2.38
Awareness	14.62 ± 3.06
Individual and societal preparedness	3.58 ± 1.32
NDL Affective Disposition Scale Total Score	47.75 ± 4.22
Disaster Preparedness Subscales	
Disaster physical protection	11.67 ± 1.97
Disaster planning	5.80 ± 0.66
Disaster assistance	6.84 ± 1.10
Disaster warning systems	3.52 ± 0.49
Disaster Preparedness Scale Total Score	27.85 ± 3.40

The relationship between nursing students’ geographic inquiry (*r*=.774, *p*=.000), personal protection measures (*r*=.924, *p*=.000), physical and ideological attitudes (*r*=.554, *p*=.000), awareness (*r*=.157, *p*=.006), and individual-societal preparedness (*r*=.128, *p*=.027) with DAO behavior is statistically significant and positive. The relationship between nursing students’ geographic inquiry (*r*=.134, *p*=.020), susceptibility (*r*=.244, *p*=.000), awareness (*r*=.830, *p*= .000), and individual-societal preparedness (*r*=.834, *p*=.000) with DAO affective disposition is also statistically significant and positive. Additionally, the relationship between nursing students’ disaster physical protection (*r*=.860, *p*=.000), disaster planning (*r*=.894, *p*=.000), disaster assistance (*r*=.633, *p*=.000), and disaster warning systems (*r*=.835, *p*=.000) with disaster preparedness is significant and positive (Table 3).

**Table 3:** Correlation matrix for NDL affective disposition, NDL behavior scale and DP scale factors for nurse students

		Geographic Inquiry	Personal protection measure	Physical and ideological attitudes	NDL Behavior Scale	Susceptibility	Awareness	Individual and societal preparedness	NDL Affective Disposition Scale	Disaster physical protection	Disaster planning	Disaster assistance	Disaster warning systems	Disaster Preparedness Scale
Geographic Inquiry	r	1												
	p	-												
Personal protection measure	r	0.571	1											
	p	0.000*	-											
Physical and ideological attitudes	r	− 0.085	0.641	1										
	p	0.141	0.000*	-										
NDL Behavior Scale	r	0.774	0.924	0.554	1									
	p	0.000*	0.000*	0.000*	-									
Susceptibility	r	− 0.014	− 0.111	− 0.113	− 0.088	1								
	p	0.809	0.055	0.050**	0.126	-								
Awareness	r	0.142	0.156	0.050	0.157	− 0.326	1							
	p	0.013**	0.007**	0.386	0.006*	0.000*	-							
Individual and societal preparedness	r	0.124	0.093	0.022	0.128	− 0.265	0.925	1						
	p	0.031**	0.106	0.708	0.027**	0.000*	0.000*	-						
NDL Affective Disposition Scale	r	0.134	0.093	− 0.021	0.104	0.244	0.830	0.834	1					
	p	0.020**	0.106	0.722	0.071	0.000*	0.000*	0.000*	-					
Disaster physical protection	r	0.019	− 0.017	− 0.018	0.000	0.031	− 0.069	− 0.052	− 0.049	1				
	p	0.744	0.771	0.761	0.996	0.597	0.234	0.371	0.399	-				
Disaster planning	r	− 0.030	− 0.068	− 0.052	− 0.060	0.051	− 0.004	0.003	0.027	0.611	1			
	p	0.607	0.240	0.370	0.296	0.373	0.945	0.965	0.641	0.000*	-			
Disaster assistance	r	− 0.033	− 0.056	− 0.050	− 0.059	0.010	0.021	0.038	0.033	0.167	0.770	1		
	p	0.567	0.334	0.386	0.310	0.862	0.711	0.509	0.567	0.004	0.000*	-		
Disaster warning systems	r	0.010**	− 0.005**	0.002**	0.006**	0.011**	− 0.083	− 0.058	− 0.072	0.730	0.657	0.424	1	
	p	0.862	0.925	0.968	0.914	0.843	0.153	0.313	0.215	0.000*	0.000*	0.000*	-	
Disaster Preparedness Scale	r	− 0.004	− 0.042	− 0.036	− 0.030	0.033	− 0.046	− 0.026	-023	0.860	0.894	0.633	0.835	1
	p	0.943	0.469	0.532	0.607	0.572	0.428	0.657	0.693	0.000*	0.000*	0.000*	0.000*	-

*p < 001, **p<.005 F

There is no relationship was found between nursing students’ natural disaster literacy behavior, affective tendencies, and disaster preparedness (Table 3).

There is no statistically significant difference was found between nursing students’ demographic characteristics and the total mean scores of natural disaster literacy scale (*p* > .05). There is no statistically significant difference was found between nursing students’ demographic dharacteristics and the total mean scores of disaster preparedness scale (*p* > .05).

## Discussion

In a study in the literature on the natural disaster literacy levels of nursing students, it was found that the students had high levels of both natural disaster literacy behaviors and natural disaster literacy affective disposition [9]. In another study, it was demonstrated that the disaster education provided to students increased the total scores of nursing students’ affective tendencies and behaviors related to natural disaster literacy [12]. According to a study by Sözcü and Aydınözü (2019) on natural disaster literacy among teacher candidates from eight universities, the teacher candidates’ knowledge and behaviors regarding natural disasters were at an intermediate level, their affective disposition were high, and their overall natural disaster literacy was at a high level [4]. Similarly, in another study by Demirdelen and Çakıcı (2021) conducted on teachers, the participants’ natural disaster literacy behavior level and affective disposition level were considered to be high [5]. Having a high level of natural disaster literacy is important as it contributes to building a disaster-resistant society and plays a crucial role in reducing the damage caused by disasters.

There are few studies in the literature that focus on determining the disaster preparedness of nurses and nursing students. Similarly to our study, in the research conducted by Baykal and colleagues (2023) with midwifery and nursing students, it was found that the students scored just above the average total score on the scale [12]. In the study by Aker and colleagues (2024) with midwifery students, similar to our study, it was found that the students’ total score on the disaster preparedness scale was slightly above the average score [13]. In a study by Pertiwi and Zakiyah (2021) with university students in Indonesia, it was found that the students’ disaster preparedness was below the average total score on the scale [14]. Research results may vary depending on different samples and the geopolitical and topographical characteristics of the country where the study is conducted. Countries located in earthquake zones, in particular, are expected to be better prepared in this regard. Although studies on disaster preparedness are limited in the literature, based on the results of existing studies, it can be said that nursing students’ disaster preparedness is not at a good level. This highlights the importance of providing nurses, who have significant responsibilities in all stages of disaster response, with the necessary knowledge and skills for disaster preparedness, especially starting from their student years through education.

In our study, it was found that as the total scores of the subscales of natural disaster literacy behavior, such as geographic inquiry, personal protection measures, and physical and ideological attitudes, increased, the nursing students’ level of developing behaviors towards natural disasters also increased. The behavioral dimension is the stage where the acquired knowledge is internalized and translated into action [15]. In this context, having a high level of the behavioral dimension is important for the internalization of knowledge and its subsequent transformation into action [16, 17]. Therefore, strengthening nursing education aimed at increasing disaster literacy should be seen as a fundamental requirement for the effective management of disasters. As the total scores of the subscales of natural disaster literacy affective disposition, such as awareness and individual-societal preparedness, increase, it can be said that the nursing students’ level of developing attitudes towards natural disasters also increases. However, in the study conducted by Şekerci and colleagues (2023), the lack of a significant difference in the “awareness” and “individual and societal preparedness” subscales after natural disaster education suggests that creating awareness to develop attitudes in natural disaster literacy is quite challenging [9]. Increasing awareness will enhance both individual and societal preparedness levels, thereby improving the levels of natural disaster literacy behaviors and attitudes.

No studies have been found in the literature examining the relationship between natural disaster literacy and disaster preparedness. The relationship between natural disaster literacy and disaster preparedness can be explained by the complementary nature of the two concepts [18]. While disaster preparedness includes physical and managerial measures directly taken against disasters, disaster literacy involves activities aimed at understanding how and why these preparations should be made [19]. Disaster literacy forms the foundation of the disaster preparedness process, as individuals and communities must first understand the nature, risks, and impacts of disasters in order to be prepared for them [20]. Individuals with high disaster literacy are better equipped with the knowledge required for preparedness, allowing them to respond more effectively during a disaster [21]. Additionally, they can take more informed steps during the post-disaster recovery process, contributing to the reconstruction of their communities. Furthermore, as disaster literacy increases, individuals’ participation in disaster preparedness also rises, which helps reduce the negative impacts of disasters on communities [22, 23]. In our study, the lack of a significant relationship between nursing students’ natural disaster literacy and disaster preparedness is thought-provoking, especially considering that the sample was taken from a university located in a region affected by an earthquake. The lack of a relationship between nursing students’ Natural Disaster Literacy and Disaster Preparedness levels may be due to several factors. Firstly, having theoretical knowledge (natural disaster literacy) does not necessarily mean that students are practically prepared for disasters; in other words, knowledge may not have translated into behavior. Additionally, disaster preparedness may depend more on experience, hands-on training, or individual motivation, whereas literacy levels might only reflect conceptual understanding. All these factors could have contributed to the absence of a statistically significant relationship between the two variables.

Integrating simulation-based training and interprofessional drills into the nursing curriculum significantly enhances students’ clinical decision-making, communication, and crisis management skills. For instance, high-fidelity simulations involving emergency scenarios encourage students to perform accurate interventions under pressure and communicate effectively within a team. Furthermore, involving nursing students in joint exercises with students from other healthcare disciplines—such as medicine, pharmacy, and physiotherapy—fosters interprofessional collaboration and role awareness.

## Limitations

The primary limitation of this study is its cross-sectional design, as data were collected at a single point in time, making it impossible to establish causal relationships between variables and providing only a snapshot of the current situation. Additionally, the study was conducted at a single university and involved only third and fourth-year nursing students, limiting the generalizability of the findings. Furthermore, the use of self-reported data poses a risk of bias, as participants’ responses may be influenced by their perceptions or social desirability, potentially affecting the accuracy of the results.

A limitation of this study is the use of self-reported data collection methods. Self-report measures are susceptible to biases such as social desirability bias and participants’ tendency to overestimate or present their behaviors in a more favorable manner. This may be particularly relevant for behavioral constructs such as disaster preparedness, where discrepancies can arise between reported and actual practices. Therefore, the findings should be interpreted with the understanding that they reflect participants’ perceptions and may not fully correspond to objectively measured behaviors.

Another limitation of this study is the lack of control for potential confounding variables that may influence both natural disaster literacy and disaster preparedness. Unmeasured factors such as demographic and experiential characteristics may have affected the observed associations; therefore, the findings should be interpreted with caution.

## Conclusion

In our study, which aimed to investigate the relationship between nursing students’ natural disaster literacy and their disaster preparedness levels, the findings revealed that while students exhibited a moderate level of natural disaster literacy behaviors, their affective tendencies toward natural disaster literacy were high. The students’ average total score on the disaster preparedness scale was slightly above the scale’s overall mean score. However, no significant relationship was found between nursing students’ natural disaster literacy behaviors, affective tendencies and disaster preparedness.

Education for nursing students on disaster literacy and preparedness should combine both theoretical knowledge and practical skills. Students should receive comprehensive training on disaster types, first aid, psychological support, basic healthcare services, and post-disaster recovery processes. Additionally, practical skills such as effective communication during disasters, crisis management, and logistical coordination should be taught. Through simulations and drills, nursing students should develop the ability to make quick and correct decisions in disaster situations. Moreover, students should be educated on the importance of raising awareness in the community about disasters and integrating this awareness into healthcare services. This training ensures that students are well-prepared for disasters and can best protect both their own safety and the health of the community.

### Implication for nursing practice

Enhancing natural disaster literacy and disaster preparedness is essential for strengthening community resilience against disasters. To achieve this, it is recommended that nursing education programs undergo a comprehensive review to integrate disaster-related content more effectively. This may include restructuring the curriculum to incorporate evidence-based disaster preparedness training and introducing elective courses focused on disaster response and management. Additionally, practical simulations, interdisciplinary collaborations, and field-based training could be integrated to bridge the gap between theoretical knowledge and real-world application. Furthermore, to ensure more robust and generalizable findings, future research should be conducted with larger and more diverse samples.

## Data Availability

No datasets were generated or analysed during the current study.
